# Risk mapping of peste des petits ruminants virus spread in nine countries surrounding the black sea: a spatial multicriteria decision analysis approach

**DOI:** 10.3389/fvets.2026.1783624

**Published:** 2026-03-16

**Authors:** Margarida Arede, Daniel Beltrán-Alcrudo, Camilla Benfield, Jordi Casal, Njeumi Felix, Giovanna Ciaravino, Alberto Allepuz

**Affiliations:** 1 Departament de Sanitat i d’Anatomia Animals, Universitat Autonoma de Barcelona, Bellaterra, Spain; 2 Food and Agriculture Organization of the United Nations Regional Office for Europe and Central Asia, Budapest, Hungary; 3 Food and Agriculture Organization of the United Nations, Rome, Italy

**Keywords:** disease spread, Eurasia, GIS-MCDA, PPR, small ruminants

## Abstract

**Introduction:**

Peste des petits ruminants (PPR) is a highly contagious viral disease of small ruminants (SR) with major socioeconomic impacts. Although targeted for global eradication by 2030, PPR continues to spread and reemerge in previously free areas. In countries surrounding the Black Sea, where small ruminant production is critical for rural livelihoods and national economies, PPR is endemic in Türkiye and has recently emerged in neighboring countries, raising concerns about further transboundary spread, including into the European Union. This study, conducted prior to the 2024–2025 outbreaks, aimed to map areas at higher risk for PPR spread following an initial incursion in nine countries surrounding the Black Sea: Armenia, Azerbaijan, Belarus, Bulgaria, Georgia, Republic of Moldova, Romania, Türkiye, and Ukraine.

**Methods:**

A spatial multicriteria decision analysis (GIS-MCDA) approach was applied using five risk factors (RFs) selected based on literature review, data availability and regional relevance: SR abundance, proportion of smallholder farming, seasonal movements of SRs, proximity to livestock markets, and proximity to previous outbreaks. Risk factor weights were derived through expert elicitation using the Analytical Hierarchy Process (AHP). A total of 18 expert responses that met consistency thresholds were used to calculate the final RF weights. Georeferenced RF data were sourced, processed and then combined with RF weights using weighted linear combination (WLC) to produce the final suitability map.

**Results:**

High risk areas for PPR spread included the majority of Türkiye, the Bulgaria-Türkiye (Thrace) border, and southern-central Georgia. Low-risk areas included Belarus, Ukraine, central and northern Bulgaria, and parts of Armenia.

**Discussion:**

This risk map can guide the prioritization of PPR surveillance, biosecurity measures, and awareness campaigns, supporting both PPRV eradication by 2030 and broader transboundary animal disease preparedness to reduce the impact of other high-priority ruminant infectious diseases.

## Introduction

Peste des petits ruminants (PPR) is a highly contagious disease caused by the PPR virus (PPRV), a *Morbillivirus* of the *Paramyxoviridae* family closely related to the rinderpest virus (1–3). PPRV is an enveloped, non-segmented, negative-sense RNA virus (1) that primarily affects domestic small ruminants (SR), namely sheep and goats, but can also cause disease in wildlife species (4). The acute form of PPR is characterized by fever, anorexia, diarrhea, ocular and nasal discharge, and erosions or ulcers in the digestive mucosae (2). Transmission occurs mainly through direct contact between infected and susceptible animals, especially via respiratory secretions during the early stages of the disease, when viral shedding is highest. Because PPRV is generally thought to be unstable in the environment, indirect transmission is less common but may occur through contact with recently contaminated materials or equipment (1, 5, 6). Animal movements, including trade, markets, and seasonal grazing, can facilitate spread between herds and across regions. Mortality and morbidity rates vary depending on host-pathogen interactions and the level of endemicity but can reach 90% in naïve populations (7, 8). Consequently, PPR poses significant threats to SR production, animal health and welfare (9), as well as the livelihoods and protein availability of SR-dependent communities.

PPR is endemic across Africa, South Asia, East Asia, and the Middle East, where nearly 2.5 billion SRs—over 80 percent of the global SR population—are kept (7, 10). Within this vast region, countries classified as “PPR-free” are at a high risk of transboundary PPR spread. The global economic impact of PPR has been estimated to range from USD 1.4 to 2.1 billion annually (11), driven not only by direct production losses but also by the extensive costs associated with surveillance and control measures. For instance, the 2018 PPR outbreak in Bulgaria incurred an estimated total cost of €2 million. Expenses were distributed as follows: direct outbreak management (11.5%), at-risk farm measures (28.2%), communication and training (18.7%), general population surveillance (24.6%), and coordination activities such as movement and border controls (17%) (Ciaravino et al., unpublished data).

Following the successful eradication of rinderpest, PPR was designated in 2015 as the next livestock disease targeted for global eradication by 2030 under the PPR Global Control and Eradication Strategy (GCES). The PPR GCES, coordinated by the PPR Secretariat—a collaborative initiative by the Food and Agriculture Organisation (FAO) and the World Organisation for Animal Health (WOAH)—focuses on strengthening veterinary services, enhancing diagnostics and surveillance systems, and implementing effective control measures such as vaccination and improved biosecurity (11). Despite these efforts, PPR has continued to spread, including into countries previously considered PPR-free. In countries surrounding the Black Sea—which spans the border between Asia and Europe, and has long been recognized as a potential pathway for the introduction of PPR into the European Union (EU)—PPR’s transboundary spread has increased in recent years (9, 10, 12). The first official report of PPR occurred in 1999 in Türkiye, where the disease subsequently became endemic (13), followed by outbreaks in Georgia in 2016 (14) and in Bulgaria in 2018 (15) that were promptly controlled (S3).

Unfortunately, in 2024, after this study was conducted the threat of PPR to Europe materialised with the first reports of the disease in Greece and Romania in July, both previously considered PPR-free by the WOAH, and later that year in November in Bulgaria (16, 17). The spread continued in 2025, with confirmed cases in Hungary in January (18), in Albania in June (19), in Kosovo in July (20) and in Croatia in December, with further outbreaks reported in early 2026 (21). These recent outbreaks highlight the urgent need for robust risk assessment tools that anticipate and mitigate disease spread.

In this scope, spatial disease modeling is employed to analyze disease risk patterns across geographic areas (22). The outputs of these models can be used to support decision-makers in prioritizing and developing effective disease management strategies (23). Although these approaches are often data driven, and their application can be limited in regions with limited surveillance coverage or in disease-free regions. In such contexts, knowledge-driven methods, such as spatial multicriteria decision analysis (GIS-MCDA) are applied. GIS-MCDA integrates existing knowledge of relevant risk factors (RFs) associated with a disease with available georeferenced data, to generate risk maps (22, 23).

The application of GIS-MCDA is particularly valuable for assessing disease risk in low- and middle-income countries, where animal disease data are often incomplete or unreliable. Contributing factors include disease underreporting, limitations of animal health surveillance systems and structural constraints within the livestock sector. Additionally, the high proportion of unregistered farms and underdeveloped national animal identification and traceability systems (NAITSs) hinder the effectiveness of disease surveillance and control programs (24).

GIS-MCDA has been used to assess the introduction and spread of PPR in South Africa (25), and East Africa (26), and it has also been applied to evaluate other transboundary animal diseases (TADs) affecting ruminants, such as foot and mouth disease (27–30) and Rift Valley fever (31–33) in Africa and Asia.

In this study, the study region includes nine countries: Armenia, Azerbaijan, Belarus, Bulgaria, Georgia, the Republic of Moldova, Romania, Türkiye, and Ukraine. This study aims to address gaps in PPR surveillance data within this region, by applying a GIS-MCDA approach to map areas at higher risk for PPR spread, assuming an initial PPR incursion. The resulting risk maps are intended to support decision-makers in planning and implementing targeted, cost-effective prevention, control, and surveillance measures. In addition, these maps can help raise awareness for PPR risk among relevant stakeholders and contribute to regional preparedness efforts.

## Materials and methods

GIS-MCDA, thoroughly described elsewhere integrates georeferenced data and value judgments (i.e., stakeholders’ preferences) to support decision-making (23, 34). In epidemiology, this method is used to map risk or suitability of disease, assisting decision-makers to prioritize risk-based disease management strategies (23, 35).

The GIS-MCDA framework involves the following sequence of steps: (1) definition of the study’s objective; (2) identification of factors and constraints associated with the objective; (3) collection of georeferenced data and geoprocessing of each factor; (4) risk factor (RF) weight elicitation and generation of the relative weight for each RF; (5) definition of the relationship between the RF and the outcome and standardization of factor data; (6) combination of the standardized RF layers and relative weights to produce a final weighted estimate of suitability for each cell in the study area; (7) uncertainty and sensitivity analysis and (8) suitability map validation (22, 23).

### Definition of the study region

**Figure 1:**
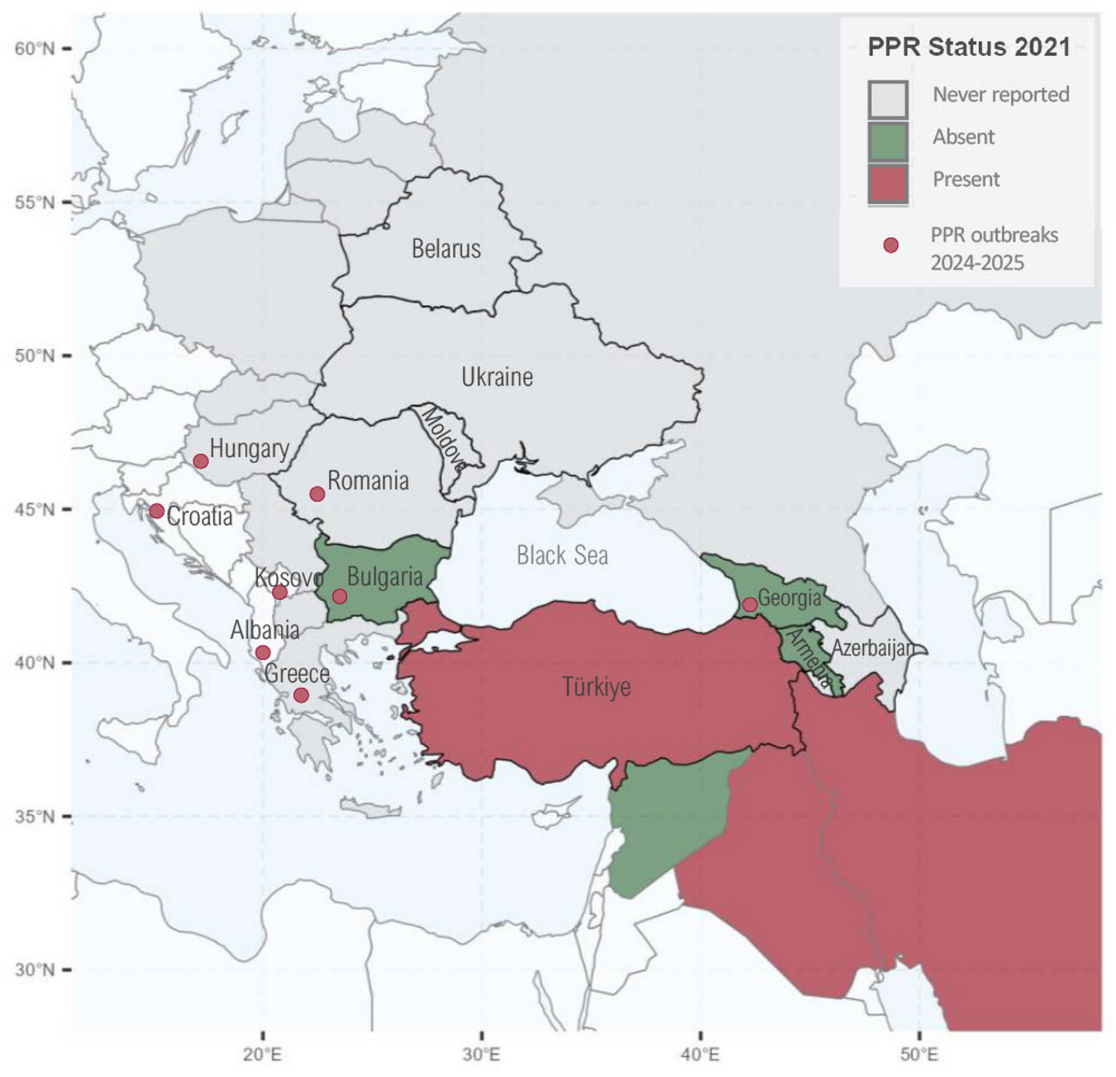
Peste des petits ruminants (PPR) status in the study region and neighboring countries in 2021. The study region—Armenia, Azerbaijan, Belarus, Bulgaria, Georgia, Moldova, Romania, Türkiye, and Ukraine—is outlined in black. Red dots indicate countries that reported PPR outbreaks in 2024 (Georgia, Greece, Bulgaria, and Romania) and 2025 (Hungary, Albania, and Kosovo). Countries without a direct border with the study region are shown in white. Maps were generated using R programming language (38).

The current study was conducted under the Europe component of a broader FAO project titled “Global Framework for the Progressive Control of Transboundary Animal Diseases (GF-TADs)”. The nine countries included—Armenia, Azerbaijan, Belarus, Bulgaria, Georgia, Moldova, Romania, Türkiye, and Ukraine—were selected based on the geographic scope defined by the donor (Figure 1).

#### Small ruminant production

The countries in the study region differed in SR population sizes and production systems. Türkiye, Azerbaijan, and Romania had the highest SR populations and per capita densities, while Belarus and Ukraine had the lowest. SR production held significant socio-economic importance in most countries, particularly in Türkiye, Azerbaijan, Georgia, Bulgaria and Romania, where it contributed to rural livelihoods, food security, and employment. In contrast, SR production played a limited role in Belarus and Ukraine, both economically and in market integration. To allow for comparison between the studied countries, a smallholding was defined as a farm that include a range of producers, from the most impoverished to those that gradually become involved with markets at a local or national level (36). This term is related to a holding with a smaller size (lower number of animal heads), often characterized by having fewer resources and low productivity, and its main purpose is subsistence or semi-subsistence. Smallholder farming predominated across the region, ranging from 76% in Bulgaria to 96% in Romania. Seasonal transhumance was common in most countries, except in Belarus and Ukraine, where sedentary systems prevailed. The role of livestock markets also varied: they were highly regulated and integrated into national monitoring systems in Türkiye and Romania, but played a minimal or informal role in others, such as Armenia, Azerbaijan, Belarus and Ukraine. In Moldova, SR production was mainly oriented toward household dairy use, with milk and cheese typically consumed by families or sold locally. In Georgia and Ukraine, SR farming was primarily carried out by rural households, reflecting a subsistence-oriented production system. During the study period, national animal identification and traceability systems (NAITS) were operational in most countries, except Armenia, Azerbaijan and Georgia, where implementation was ongoing or newly introduced (37).

#### PPR status, surveillance and control

PPR status varied across the study region. Türkiye was endemic in Anatolia but maintained a PPR protected status in Thrace since March 2021. Georgia and Bulgaria experienced outbreaks in 2016 and 2018, respectively, which were quickly contained through stamping out, movement restrictions, and—in Georgia—vaccination. Georgia also implemented ongoing risk-based surveillance, awareness campaigns, and annual vaccination of young SRs until 2022 (12). Bulgaria and Georgia reported new cases in 2024, and Romania confirmed its first outbreak in 2024. Armenia, Azerbaijan, Belarus, Moldova, and Ukraine remained officially PPR-free as of mid-2025. Among neighboring countries, Iran and Iraq are endemic, and Greece, Hungary and Albania—previously free of PPR—reported their first outbreaks in 2024, 2025, and 2025, respectively. Figure 1 depicts the PPR status of the study region in 2021 and indicates countries with outbreaks in 2024 and 2025. PPR surveillance and control also varied across the study region. Active or risk-based surveillance was implemented in Armenia, Azerbaijan, Georgia, Romania, and in the Southeast region of Bulgaria, which borders Türkiye-Thrace, while Moldova and Ukraine relied on passive surveillance. Belarus did not include PPR in its national surveillance program. In Türkiye, surveillance was passive in Anatolia, and since 2021 it was active in Thrace as part of its PPR-protected status. Vaccination was applied only in Anatolia (Türkiye) and in Georgia, following the 2016 outbreak, while it was not applied in the remaining countries of the region.

### Definition of objective

This study aims to identify high-risk areas for PPR spread in the participating countries, assuming an initial introduction of the virus, and validate the resulting suitability map using 2020–2021 PPR outbreak data from Türkiye.

### Identification of risk factors

The RFs associated with the risk of PPR spread were identified through an extensive literature search conducted on Google Scholar and PubMed. The search used the terms “PPR” OR “peste des petits ruminants”, AND “risk mapping”, OR “spatial risk”, OR “risk factors”, focusing on studies published between 2010 and 2020. The risk factors included in the study were selected by all the authors based on their consensus in the literature search, their importance in the context of small ruminant production in the study region, and the availability of geo-referenced (spatial) data or proxy data across all countries. The selected RFs and their associated hypotheses are detailed in Table 1. Additionally, an overview of the key characteristics of the SR sector and the PPR status and management activities in the studied countries are shown in Supplementary Materials S1, S2.

**Table 1:** Selected risk factors (RFs) and associated hypothesis with the PPR spread.

Risk factor	Associated hypothesis
Small ruminant (SR) abundance	Sheep and goat abundance is associated with a higher risk of PPR spread (26, 73–76), as PPR virus transmission is more likely to occur when there is a higher contact rate between susceptible and infected animals and/or their fresh secretions or feces (9).
Proximity to areas previously affected by PPR outbreaks	Areas previously affected by PPR outbreaks (or outbreaks of similar TADs) have been associated with a higher risk of PPR spread (or of similar TADs) (9, 28, 59). This increased risk may reflect the presence of uncontrolled animal movements (including informal and illegal trade), and lower biosecurity levels.
Proportion of smallholder farming	Smallholding production has been associated with a higher risk of PPR spread (25, 77–79). This production type is associated with traditional practices and limited management capacity, which can hinder disease control and prevention efforts. In some countries, it involves communal animal keeping, leading to increased contact between SR from different herds, and a higher risk of disease spread.
Proximity to live animal markets	Live animal markets have been associated with PPR spread (26, 80–83), as they promote SR gatherings from different herds, regions, or even countries, and facilitate direct contact between them. We hypothesize that farms located closer to a livestock market are more likely to trade animals in these markets, and the surroundings are therefore associated with a higher risk of PPR spread.
Seasonal pastures	Seasonal movements to pastures (78–80, 83–88) have been associated with a heightened risk of PPR spread. This practice facilitates direct contact between animals from different herds, regions, or even neighboring countries, on shared grazing areas and water points. Or indirect contact with fresh secretions or feces on pastures, water points, and animal shelters.

### Collection of georeferenced data and geoprocessing of RF layers

Small ruminant abundance data was sourced in raster format from the Gridded Livestock of the World (GLW4) dataset (38, 39), modified using national sheep and goat census data obtained from participating countries and adjusted to FAOSTAT 2020 data. This raster layer was used to create a template to rasterise the other RF layers.

All remaining georeferenced factor data—including SR seasonal movements (except for Romania), proportion of smallholdings, and livestock market locations—were provided by designated national consultants from each participating country. Additionally, data for seasonal movements of SR in Romania was obtained through a literature review at regional level (40–43). Georeferenced data for each risk factor was pre-processed using QGIS (projection: ETRS89-extended/LCC Europe, EPSG:3034), and rasterised using R programming language (44) to match the raster template resolution of 0.0833 by 0.0833 decimal degrees (approximately 10 by 10 km) in World Geodetic System 1984 (WGS_84).

The RF “proximity to previous outbreaks” was derived from a total of 395 georeferenced PPR outbreaks reported to the World Animal Health Information System (WAHIS) between 2016 and 2019 (10 from Bulgaria, 1 from Georgia, 384 from Türkiye). The RF “proximity to livestock markets” was based on 324 georeferenced livestock markets located across our study region. Both proximity-based RFs were processed in QGIS using the “distance to nearest Hub (points)” tool, to calculate the Euclidean distance from 10 by 10 km grid centroids to the nearest PPR outbreak or livestock market.

The RF “proportion of smallholdings” was derived either directly from national census data or, where unavailable, calculated as the proportion of SR smallholder farms within each administrative area. The administrative level used varied across countries, due to differences in data resolution and availability, wherever possible the lowest available level was used. In Türkiye, due to a lack of disaggregated data by production system, a constant national estimate was applied. In Armenia, Azerbaijan, and Georgia, the absence or recent implementation of National Animal Identification and Traceability Systems (NAITS) meant that decentralized and non-standardized records were used.

Regarding the RF “seasonal movements”, systematic records were not consistently maintained across most countries of the study region, and as a result, the data provided varied in format. These formats comprised text descriptions of animal movements to pastoral sites, annotated maps indicating the origin and destination of SR, and quantitative data for pastoral movements at province or farm level. To ensure comparability, the qualitative data were converted into numerical values for the smallest administrative area available in each country. These were then collated with the quantitative movement data and rasterised. The resulting RF layer illustrates the number of administrative areas of origin that relocated animals to pastures for each respective administrative area.

### Risk factor weight elicitation

Opinions were gathered using a pairwise comparison matrix exercise (S4), hereafter referred to as the elicitation exercise, which is based on Saaty’s analytical hierarchy process (AHP) (45). In brief, AHP systematically evaluates preferences by breaking down complex decisions into a structured hierarchy. Through pairwise comparisons, it quantifies subjective judgments to create priority scales. This approach enhances consistency and transparency in decision-making.

To complete the elicitation exercise (EE), we recruited individuals from the participating countries with knowledge of the SR sector in the study region and PPR international experts. Participation in the exercise was voluntary. Participants were divided into two expert groups: (1) national experts, comprising academics and veterinary authorities from participating countries; and (2) international PPR experts from academic or research institutes, as well as representatives from the PPR Secretariat, the PPR Global Research and Expertise Network (PPR-GREN), FAO and WOAH.

The EE was developed using Microsoft Office Excel (2019) and paired with a supplementary document detailing the project’s background and instructions for the exercise. Both documents were prepared in English and then translated into Russian, for participants from countries where Russian is widely spoken. The participants were familiarized with the exercise during a regional training workshop focused on risk-based approaches for PPR prevention, control, and eradication The workshop was organized by the FAO Regional Office for Europe and Central Asia (FAO-REU), the PPR Secretariat, and the Autonomous University of Barcelona (UAB). It was conducted online, over 3 days (on 31 January, 2, and 4 February 2022), in English with simultaneous interpretation into Russian. Attendees comprised all country experts and some international experts who were sent the EE and supplementary document during the workshop. Afterward, the same documentation was emailed to other selected experts, primarily affiliated with the PPR Secretariat’s network.

This exercise aimed to determine experts’ opinions on the relative importance of the selected RFs for PPR spread within the study region. Participants were asked to compare the RFs in pairs selecting one of nine expressions from a predefined sequential key. This key ranged from “extremely less important”, to “extremely more important”, with equivalent as the midpoint (S4). After the EEs were completed, the qualitative expressions were converted into corresponding numerical values (S4) for further analysis.

### Generation of risk factor weights

The RF weights attributed by each expert were derived from the individual EE, following the AHP calculation steps (S5). After converting the key expressions into their respective numerical values, we calculated the normalized eigenvector by taking the n^th^ root product of each row and dividing it by the total sum. The normalized eigenvector provides an approximation of the weight attributed to each risk factor (29).

To ensure the reliability of expert judgments and avoid the inclusion of randomly or inconsistently filled matrices, we calculated the consistency ratio (CR) for each expert’s response (46). The CR measures the internal coherence of the pairwise comparisons, meaning how consistent an expert’s judgments are when comparing multiple criteria, with lower values indicating higher consistency. A perfectly consistent matrix has a CR of 0, while higher values suggest logical contradictions or inconsistent ranking of risk factors. Following (47), responses with a CR ≥ 0.14 were considered inconsistent and excluded from the analysis, as they may reflect random or illogical judgments. Because the CR is calculated for the entire pairwise comparison matrix rather than for individual comparisons, the whole contribution from experts exceeding this threshold were excluded. Only matrices with CR < 0.14 were retained to calculate average RF weights for national and international expert groups, as well as an overall average. An example of CR calculation and an inconsistent response is provided in S5.

### Standardization of risk factor layers

RF layers were standardized to a common continuous scale between 0 and 1 using fuzzy membership functions (19). The shape (e.g., sigmoidal or linear) and direction (e.g., increasing or decreasing) of the membership function assigned to each RF layer reflect its association with the risk of PPR spread in the region (34). Function thresholds were assigned to minimum and maximum RF raw values or a predefined limiting value. For this study, the membership function for the standardization of each RF was selected based on literature using similar RFs to estimate the spatial risk of PPR spread or other TADs (26, 28, 29, 31). Standardized RF layers were generated by applying the membership function to the range of RF raw values falling within the specified thresholds (23, 34). Standardization of rasterised factor layers was computed in R programming language (44) using a code adapted from (25) with a final resolution of 0.0833 by 0.0833 decimal degrees (approximately 10 by 10 km) (Table 2).

**Table 2:** Selected risk factors (RFs), minimum and maximum thresholds, and the membership function applied for the standardization of each RF layer.

Risk factors	Minimum value	Maximum value	Membership function
Small ruminant abundance	0	Max SR heads/pixel	Increasing linear monotonical
Proximity to PPR outbreaks	0	50 km radius	Sigmoidal Decreasing - between 0 and 50 km, >50 km negligible risk
Proportion of smallholder farming	0	1	Increasing linear monotonical
Proximity to live animal market	0	50 km radius	Decreasing linear monotonical - between 0 and 50 km, >50 km negligible risk
Seasonal pastures	0	1	Binary

### Combination of the spatial layers and creation of a suitability map

To create the final suitability maps, a weighted linear combination (WLC) (48) (Equation 1) was applied to integrate the standardized RF layers and final average RF weights. The suitability for PPR spread was conveyed on a continuous scale ranging from 0 (unsuitable) to 1 (totally suitable).


$$\text{Suitability index}=\sum_{i=1}^{n}w_{i}\times RF_{i},1\leq i\leq n$$ (1)

n is the number of RFs, w_i_ the weight and RF_i_ the pixel value of RF i.

Three suitability maps were generated: one using the average of RF weights from all consistent matrices (CR < 0.14), and the other two applying the average of the RF weights attributed within the predefined expert groups, national and international experts.

### Validation

We validated the final suitability map in Türkiye with all PPR outbreaks reported in this country in 2020 and 2021 (90 outbreaks). For this purpose, we applied the receiver operating characteristic (ROC) analysis (49), and calculated the area Under the Curve (AUC), with the package *pROC* using R language. Since no absence data was available from active or field surveillance, pseudoabsence points were randomly generated at the same ratio as outbreak data, under the condition of being located at a minimum of 25 km distance from either an outbreak point or another pseudoabsence.

### Sensitivity and uncertainty analysis

Sensitivity analysis was done by applying the one-at-a-time (OAT) method (50). In this process, the values of the factor layer (input), are changed one at a time, to evaluate the effect on the change of the suitability index (output). For the proposed framework (51), we set a stepwise change of 1%, with a ± 25% range, to the mean weight of each RF. The weights of all the other RFs were adjusted proportionally to ensure the sum of RF weights was 1. This process generated 250 alternative suitability maps for PPR spread in the region, with the results presented in a graph showing the mean of the absolute change rate (MACR), which represents the average proportion of change in suitability values across all 250 simulations. Thus, the original suitability map (with the original RF weights) was compared quantitatively for all pixels to the alternative simulated maps. Thereafter, uncertainty values were derived from the standard deviation of the alternative maps generated through the sensitivity analysis. These values, presented in a map of the region, depict the uncertainty surface associated with the method applied (52).

### Spatial agreement of 2024–2025 PPR outbreaks with final suitability map

To assess the spatial agreement between PPR outbreaks occurring in 2024 and 2025 and areas of high suitability index for PPR spread, we overlaid the georeferenced locations of reported PPR outbreaks in the study region—1 in Bulgaria, 1 in Georgia, 67 in Romania, and 20 in Türkiye—with the final suitability map generated in an earlier step through GIS-MCDA.

Suitability index values at each outbreak location were extracted using the extract() function from the raster package in R (44). For each affected country, descriptive statistics (number of outbreaks, mean, minimum, and maximum suitability values) were calculated to evaluate the degree of spatial agreement between model predictions and observed outbreak locations.

## Results

### Weight calculation and suitability map

A total of 39 elicitation exercises were completed and returned by the participants. Of these, 18 with a consistent ratio (CR < 0.14) were retained and assigned to the respective participating group: national or international experts. The remaining 21 responses were excluded due to higher inconsistency (CR ≥ 0.14), which for example, reflected logically contradictory pairwise comparisons—such as ranking A > B, B > C, but then C > A—or assigning equal importance to nearly all risk factors, suggesting little discrimination between them. The number of consistent and not consistent responses for national experts (by country) and international experts is shown in S6.

Of the retained responses, eight were from international experts and 10 from national experts. Among the national experts, three consistent responses were from Türkiye, two each from Armenia and Azerbaijan, and one each from Bulgaria, Georgia, and Moldova.

Table 3 presents the RF average, minimum, and maximum weights calculated for the total number of consistent responses within each expert group. Among international experts, the highest weight was attributed to the *proximity to PPR outbreaks*, followed by *small ruminant abundance*, and the least weight to *proximity to live animal markets*. National experts assigned the highest weight to *small ruminant abundance*, followed by *proximity to PPR outbreaks*, with the lowest weight given to the *proportion of smallholder farming*.

**Table 3:** Risk factor (RF) weights: average, minimum, and maximum weights (indicated in square brackets) attributed by all experts, and per expert group.

Risk factor	Weight		
	Total (*n* = 18)	International experts (*n* = 8)	National experts (*n* = 10)
Small ruminant abundance	0.31 [0.08, 0.53]	0.23 [0.08, 0.48]	0.37 [0.09, 0.53]
Proximity to PPR outbreaks	0.29 [0.03, 0.62]	0.30 [0.03, 0.62]	0.28 [0.03, 0.45]
Proximity to live animal market(s)	0.13 [0.03, 0.28]	0.12 [0.03, 0.28]	0.13 [0.04, 0.28]
Proportion of smallholder farming	0.14 [0.04, 0.50]	0.18 [0.04, 0.50]	0.11 [0.04, 0.18]
Seasonal pastures	0.14 [0.02, 0.56]	0.16 [0.02, 0.56]	0.12 [0.03, 0.40]

**Figure 2:**
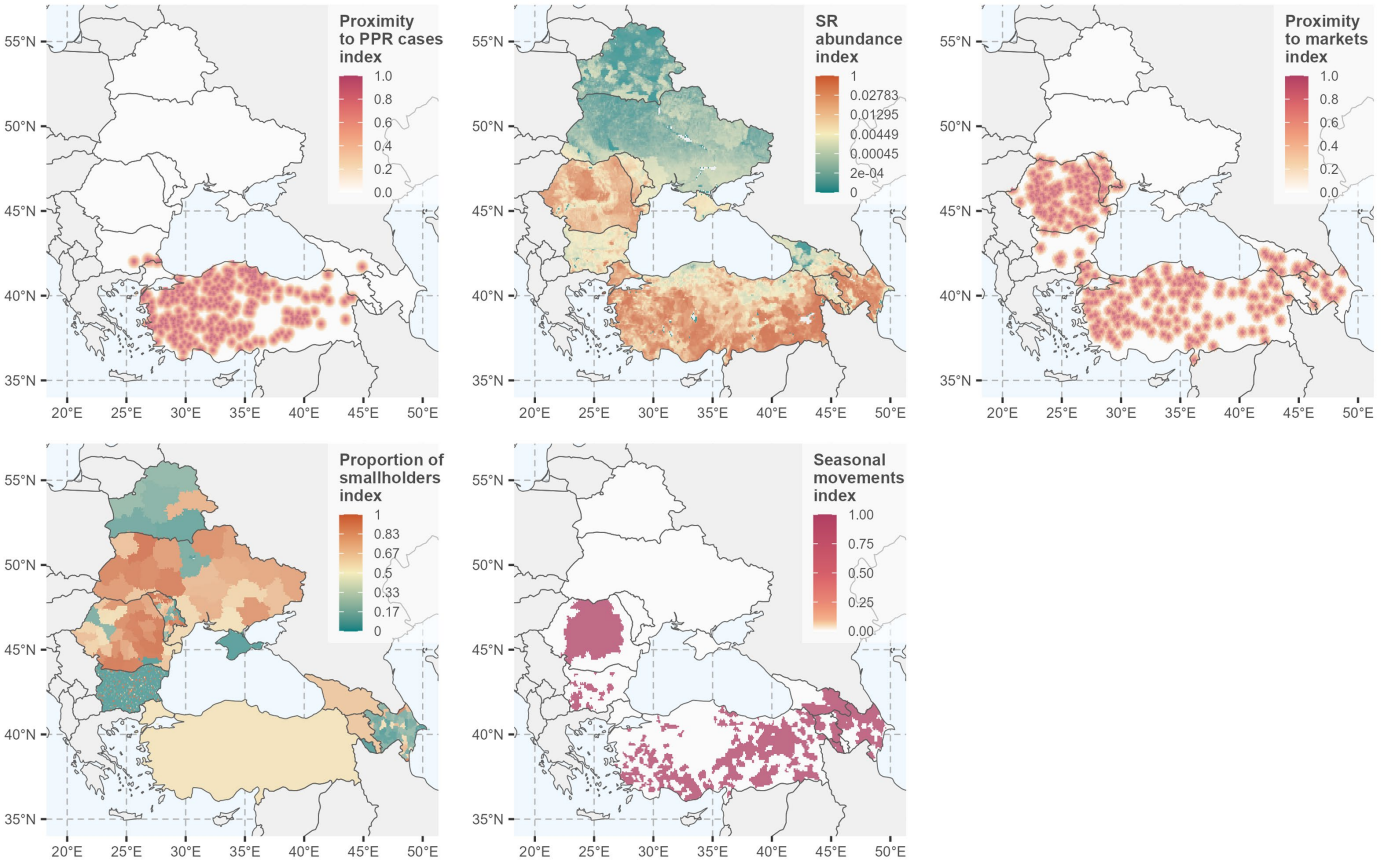
Standardized spatial peste des petits ruminants (PPR) suitability indices for the risk factors (RF) applied in the modeling approach.

**Figure 3:**
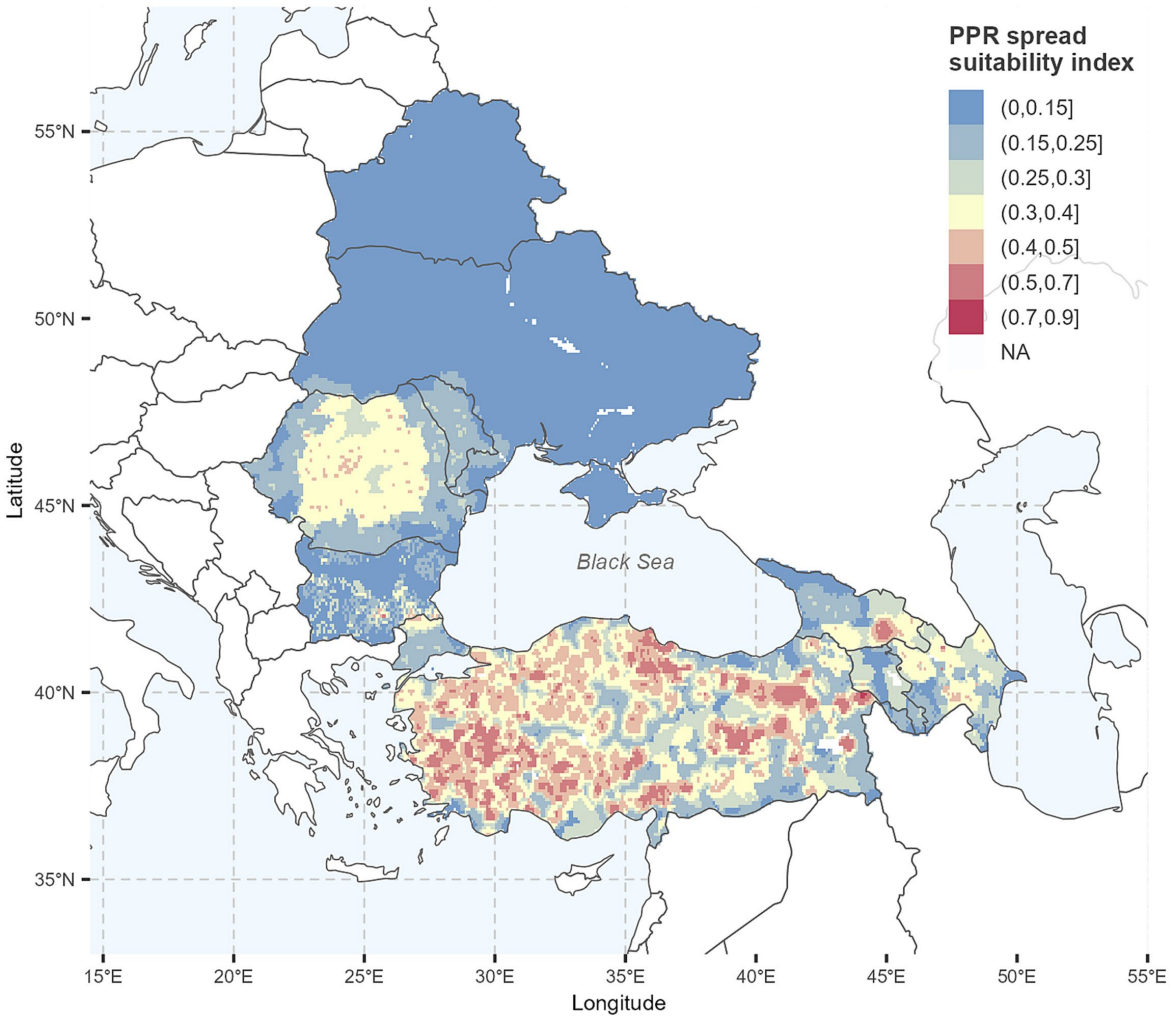
Suitability map for the spread of peste des petits ruminants (PPR) in small ruminants (SR) and location of PPR outbreaks reported between 2020 and 2021 in the study region. Maps were generated using R programming language (38).

The resulting standardized RF layers are shown in Figure 2. The final suitability map for PPR spread (Figure 3) was generated using the mean RF weights from the 18 consistent expert elicitation responses. The suitability index (SI) is displayed using a diverging spectral color scale, categorized into ranges, where blue and green (below 0.3 SI) indicate low suitability, yellow and light orange (between 0.3 and 0.5 SI) indicate medium suitability, and darker orange and red (above 0.5 SI) indicate high suitability for PPR spread. Suitability maps based on RF weights from national and international expert groups are presented in S7. Hereafter, suitability index is referred to as suitability of PPR spread for consistency and clarity.

We observed high suitability for PPR spread throughout Türkiye (eastern to center Anatolia, interior western Anatolia, and close to the Armenian border), on the border of Bulgaria with Türkiye (Thrace), and in southern-central Georgia. The lowest suitability was observed in Belarus and Ukraine, across west, center, and northern Bulgaria, and across Armenia.

### Validation

Validation was performed using PPR outbreak data from Türkiye reported in 2020 and 2021, noting that the georeferenced outbreaks for the suitability map RF ‘Proximity to PPR outbreaks’ were from 2016 to 2019. The ROC AUC associated with the suitability map for the PPR spread demonstrated the capacity of the model to distinguish “presence” from “pseudoabsence” with good predictive accuracy [AUC = 74.2%; 95% CI (66.9–81.5%)]. ROC curves for the three suitability maps are presented in S8.

### Sensitivity and uncertainty analysis

The global sensitivity analysis depicted the MACRs for each factor, for which higher absolute values have a positive correlation with its sensitivity. The graph shows that *proportion of smallholder farms* had the highest RF sensitivity, followed by *small ruminant abundance*, *distance to previous PPR outbreaks*, *seasonal movements*, and finally, *distance to markets* (S9).

**Figure 4:**
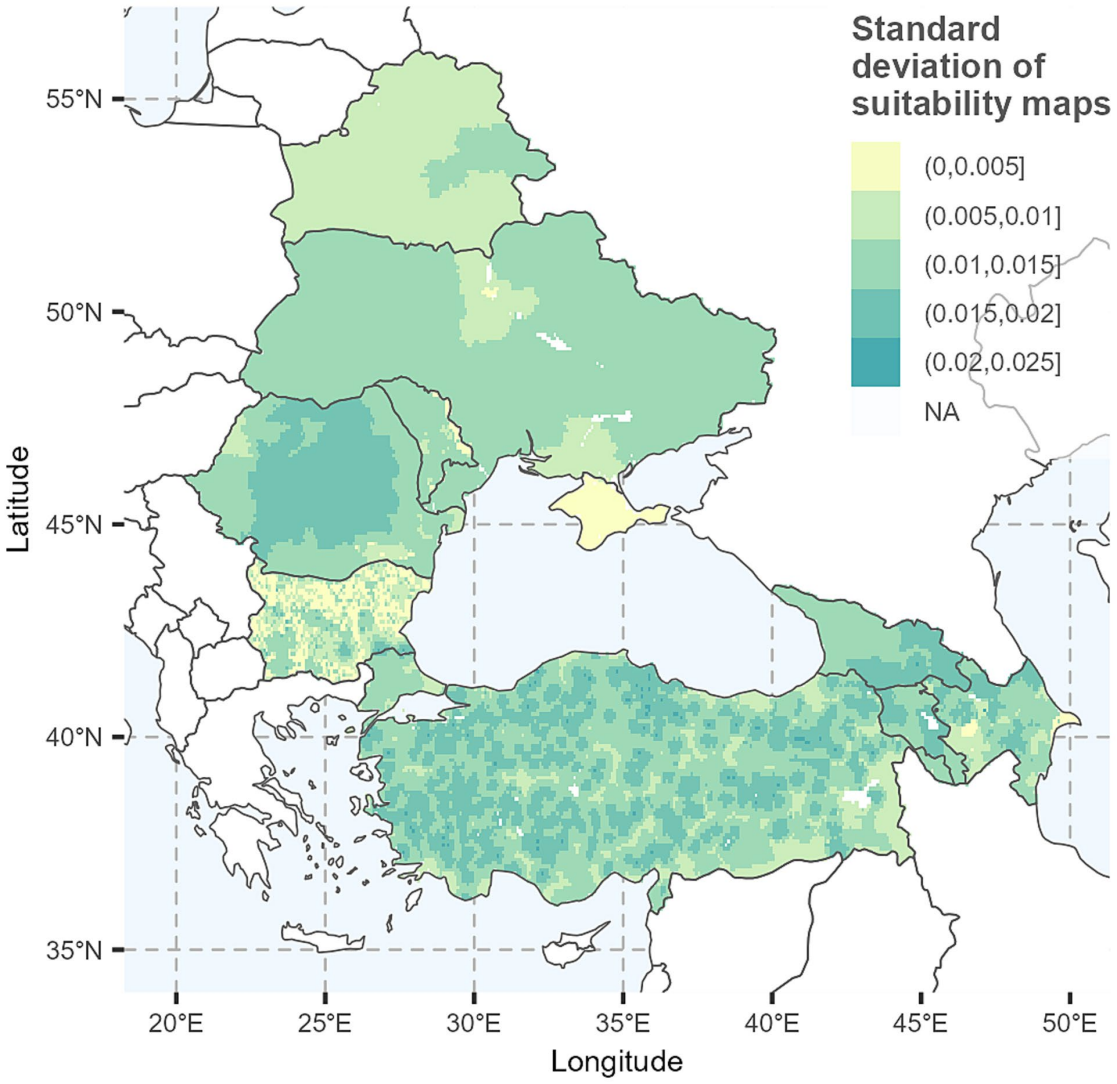
Uncertainty map. The map shows the standard deviation of the suitability maps for peste des petits ruminants (PPR) spread in small ruminants (SR) in the study region. Maps were generated using R programming language (38).

The uncertainty map, illustrated in Figure 4, based on 250 maps with adjusted weights, shows a maximum standard deviation value lower than 0.025, indicating that the risk map predictive ability was stable when RF weights changed.

### Recent PPR outbreaks in the study region and neighboring countries and overlay with the suitability map

**Figure 5:**
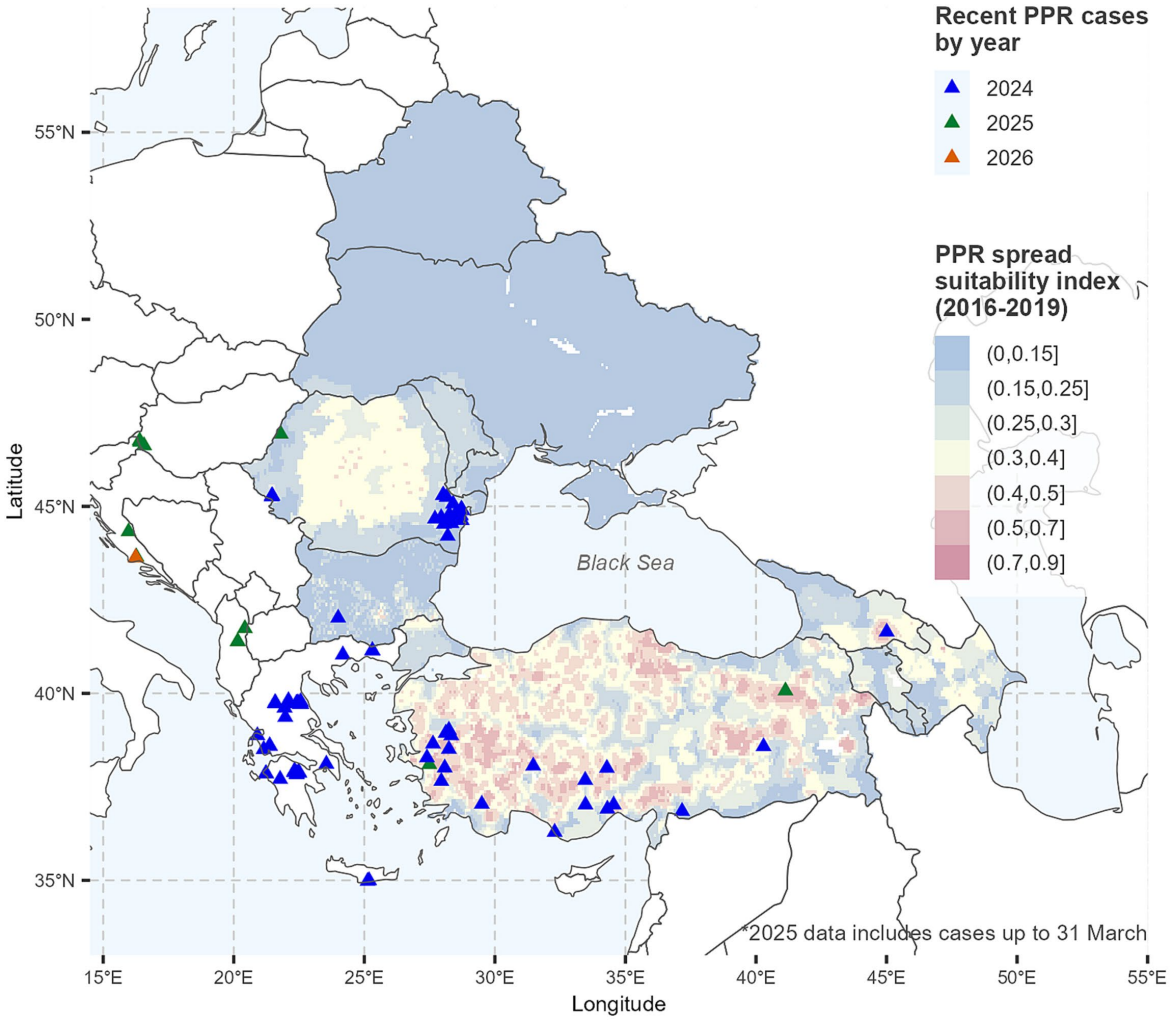
Suitability map for the spread of peste des petits ruminants (PPR) in small ruminants (SR) and location of 2024–2025 PPR outbreaks in the study region. Maps were generated using R programming language (38).

A visual comparison of the final suitability map with newly reported PPR outbreaks in 2024–2025 (Figure 5) depicted clear spatial differences between endemic and the newly affected countries in EU. In Türkiye and Georgia, both with prior PPR outbreaks, new cases occurred in areas predicted as highly suitable for PPR spread. In contrast, outbreaks in Bulgaria and Romania showed limited spatial overlap with high-suitability zones. These trends are supported by the extracted suitability index values, which were higher in Türkiye (mean = 0.41) and Georgia (0.56), compared to Romania (0.19) and Bulgaria (0.14) (Table 4).

**Table 4:** Number of reported PPR outbreaks and summary statistics of suitability index values at outbreak locations in 2024–2025.

Country	Number of reported outbreaks	Suitability index Mean [Min-Max]
Bulgaria	1	0.14
Georgia	1	0.56
Romania	68	0.19 [0.15–0.23]
Türkiye	20	0.41 [0.15–0.62]

Suitability values were extracted from the suitability map using R (38).

## Discussion

In this paper, we evaluated the spatial suitability for PPR spread in nine countries surrounding the Black Sea using a knowledge-driven approach. Despite sustained global and regional efforts toward PPR eradication by 2030, PPR remains a transboundary threat. Since 2024, multiple outbreaks have been reported within the study region (e.g., Georgia, Bulgaria, and Romania), as well as in neighboring countries reporting PPR for the first time, highlighting the need for continuous risk assessment.

Our findings show that suitability for PPR spread is consistent with known PPR distribution in this region. High suitability was found in areas where PPR was endemic (Türkiye), and in regions with outbreaks in the past decade, such as central Georgia and southeastern Bulgaria. Belarus and Ukraine, where PPR has never been reported, exhibited the lowest suitability for PPR spread, likely due to their lower sheep and goat populations and the minor socioeconomic role of the small ruminant sector. Additionally, SR were not commonly traded in livestock markets, and seasonal movements to pastures were uncommon, resulting in a negligible contribution of these risk factors to the suitability map.

Among the selected RFs, *SR abundance* and *proximity to previous outbreaks* were top-ranked by both national and international experts, however, the order of their importance differed. National experts ranked *SR abundance* as the most important factor, while international experts prioritized *proximity to previous outbreaks*. This aligns with known variations of expert opinions depending on professional background, converging with a study using a similar methodology (25).

Concerning data quality, the top-ranked RFs were based on uniform, high-resolution datasets available across the region. *SR abundance*, in particular, was preferred over farm or animal density, as it was freely available and extracted from a highly reliable and high-resolution dataset (53). Other RFs relied on data provided by the participating countries with variable quality, reflecting common constraints in transboundary disease risk mapping in low resource settings. While not ideal, this approach aligns with previous GIS-MCDA studies in similar settings, where detailed spatial data on key RFs are often lacking (23, 31, 54). In particular, the RFs *seasonal movements* and *proportion of smallholders* were derived from heterogeneous data sources due to differences in data availability, recording systems, and privacy restrictions across countries. In Armenia, Azerbaijan, and Georgia, the absence or recent implementation of NAITS (55–57) led to reliance on unstandardized recording methods, which may have affected estimates the number of smallholder. In Türkiye, information on farm production purposes was unavailable; therefore, the proportion of smallholders—assumed by national experts to be relatively uniform across the country—was applied at a national level. These data limitations also restricted the availability of direct measurements for some key transmission processes.

When direct measurements of known RFs for PPR spread were unavailable, proxy data were used. For example, live SR movements— an important driver of long-distance infectious disease spread (58)—were proxied using *proximity to livestock markets* and *seasonal movements*, as in previous studies (25, 26). While these variables identify areas where animals are likely to mingle, they do not fully capture the complexity of SR movement networks.

Similarly, *proximity to previous PPR outbreak*s served as a proxy to areas with uncontrolled animal movements (including informal and illegal trade) and lower biosecurity levels—an approach used in similar GIS-MCDA studies (28, 59). However, its relevance may vary throughout the region, showing regional variability due to several factors: (1) the inclusion of outbreak data from both endemic (Anatolia) and non-endemic areas (Bulgaria and Georgia) with different disease dynamics; (2) varying national surveillance capacities that may affect outbreak reporting completeness; and (3) differences in control measure implementation (e.g., vaccination is implemented in Anatolia). Despite these limitations, this RF was retained due to the current unavailability of spatially explicit data on informal livestock trade or biosecurity levels. Furthermore, other factors, such as wildlife interactions or husbandry practices, were omitted due to a lack of standardized georeferenced data, a common limitation in transboundary animal disease risk mapping (22, 54, 60).

Overall, while the suitability map provides a practical starting point for assessing PPR spread in the region, its outputs should be interpreted with caution in areas where epidemiological contexts diverge and data limitations persist. In particular, knowledge-driven GIS-MCDA approaches inherently involve a degree of subjectivity associated with expert opinion elicitation (22, 54).

In this study, the use of individual pairwise comparison matrices allowed the assessment of response consistency and comparison of RF weights between national and international experts. To reduce randomness and retain reliable responses, matrices with a consistency ratio below 0.14 were excluded, following established recommendations (61–63). Differences in risk factor prioritization between expert groups likely reflect not only professional background but also regional and epidemiological context, as study countries vary widely in small ruminant production systems, socio-economic conditions, and disease management capacities. Additionally, the relatively high response exclusion rate suggests that AHP exercises involving multiple or closely related RFs can be cognitively demanding for participants, which may increase inconsistency despite careful instructions. Alternative approaches aimed at achieving consensus (e.g., Delphi or focus-group methods) could be applied in future work to involve a larger number of participants and to derive more robust or region-specific RF weights for individual countries or groups of countries with similar characteristics (64–66).

In this study, the suitability map was validated using georeferenced PPR outbreak data from Türkiye, a step often omitted in spatial risk mapping due to limited data availability. The model showed acceptable predictive performance (74.2, 95% CI: 66.9–81.5%) (67). However, this validation is primarily applicable to Türkiye, as extending it to other countries would require access to comparable outbreak datasets. The predictive performance may also have been influenced by the high weighting assigned to the risk factor “proximity to previous outbreaks,” derived from the 2016–2019 period when PPR notifications peaked in Türkiye. This peak was followed by a marked decline in reported outbreaks, likely reflecting effects of control measures, lifelong PPR immunity developed in vaccinated and surviving animals, herd renewal rates, and strengthened surveillance and movement controls implemented to prevent PPR reintroduction into the EU (10), in support of Thrace’s PPR zonal freedom in 2023 (12, 68).

In addition, the spatial heterogeneity observed in the uncertainty map, where areas with higher suitability also showed greater uncertainty, likely reflects the combination of high-weighted RFs and high input values (values within the RF layer) within the same locations. When multiple RF with higher influence occur simultaneously, even small adjustments in weighting can disproportionately affect the final suitability index. Consequently, uncertainty appears in areas identified as high risk, indicating that predictions in these areas are more sensitive to the choice of weights and should therefore be interpreted with caution.

Finally, visual comparison of the most recent 2024–2025 PPR outbreaks with the suitability map revealed contrasting patterns between endemic and newly affected areas. Recent outbreaks in Türkiye were consistent with areas of high suitability for PPR spread, whereas outbreaks in Bulgaria and Romania showed poor concordance with predicted risk. This discrepancy has important implications in targeting PPR surveillance and control. In endemic regions, our risk mapping appropriately identifies areas of persistent transmission risk, where conventional control measures targeting known livestock movement patterns could be effectively applied. In contrast, the model’s lower predictive ability in newly affected areas (Bulgaria and Romania) highlights the likely importance of informal and illegal SR trade, particularly movements associated with cultural or religious events during periods of increased animal trading (69, 70). These informal transmission pathways are missed by official surveillance systems, creating significant gaps in risk assessment for regions experiencing initial PPR incursions.

These limitations could be addressed by integrating community-based surveillance that engages local stakeholders to document informal animal movements and culturally rooted practices that may contribute to PPR spread. Participatory approaches such as participatory mapping, and local reporting networks can complement conventional surveillance by enabling earlier detection, improving risk assessments, and supporting control strategies tailored to regional transmission dynamics (71). The need for such integrated surveillance is further underscored by recent epidemiological and genomic evidence from the 2024–2025 European incursions, indicating that these outbreaks share a common origin and are linked to strains circulating in North and East Africa with documented epidemiological connections between Romania and Greece and continued spread to additional countries (72). Together, these findings illustrate the dynamic nature of PPR transmission across the region and emphasize the value of periodically updating suitability assessments to maintain effective risk prioritization and preparedness in newly affected areas.

Beyond its immediate relevance for PPR, the approach applied in this study could be refined for surveillance and control of other TADs affecting small ruminants. In this study, apart from *proximity to previous PPR outbreaks*, all the risk factors used, are commonly associated with the spread of other diseases (e.g., foot-and-mouth disease, sheep and goat pox, and brucellosis). As a result, our suitability map can also inform preparedness, surveillance, and control strategies beyond PPR, especially in Türkiye, where it was validated. This aligns with the broader goal of the PPR Global Control and Eradication Strategy, which emphasizes the need to strengthen veterinary services and reduce the impact of multiple high-priority small ruminant diseases.

## Conclusion

Despite the limitations associated with spatial knowledge-driven methods such as GIS-MCDA, these can be useful tools to study disease dynamics and data gaps in resource limited settings. The resulting risk map was validated for Türkiye and provides insights for national authorities in this country to strategically target their interventions in high-risk areas. In these areas, risk-based interventions may include targeted PPR vaccination (in Anatolia), enhanced passive surveillance and, when feasible, active surveillance to support early disease detection. Complementary efforts include training and awareness campaigns toward key stakeholders within the small ruminant value chain, such as field veterinarians, farmers, middlemen, and individuals working at livestock markets and slaughterhouses. These efforts aim to improve both internal and external biosecurity while stressing the importance of timely disease reporting.

As the 2030 deadline for PPR eradication approaches, it is critical for countries that have never reported PPR or are officially recognized as PPR-free by WOAH to remain vigilant and maintain robust contingency plans to prevent disease introduction and spread.

## Data Availability

The datasets presented in this article are not readily available for public access due to restrictions imposed by the governmental entities of the nine participating countries. The data that support the findings of this study were used under license from these entities and are subject to confidentiality and legal constraints. Requests to access the datasets should be directed to mcastroarede@gmail.com.
