# Supplementary material for: Risk mapping of peste des petits ruminants virus spread in nine countries surrounding the black sea: a spatial multicriteria decision analysis approach
Source: Front Vet Sci. 2026 Mar 16;13:1783624. doi: 10.3389/fvets.2026.1783624 (PMC13033527; doi:10.3389/fvets.2026.1783624)
Supplement: Supplementary file 1 [file Supplementary_file_1.pdf]

Supplementary Materials

S1: Key characteristics of the small ruminant (SR) sector in the study countries.

**Country**	**Human population (thousand)**	**GDP/ capita 2020 (US$)**	**Agriculture to GDP (%) (2015-2020)**	**Livestock production for agricultural GDP (%)**	**Employment in agriculture (%)**	**SR population (2021)**		**Production types**		**Animal identification and traceability system (Ruminant ID/ Farm ID)**	**Seasonal movements**	**Markets**	**Notes**
						Heads (thousand)	% SR in region	Smallholder farms	Commercial farms				
**Armenia**	2.963	4.623	17,2-**11,7**	32%	42%	**690** (0.23 hds/capita)	**0,9%**	95%	5%	Under development/ implementation	SR are sent to pastures during spring-summer and are kept in stables during autumn-winter.	Livestock markets play a minor role in SR trade. Most farmers sell animals directly to consumers or traders without market infrastructure.	The livestock sector contributes significantly to national food security and provides draught power and organic fertiliser. Small ruminants are grazed in pastures during spring and summer and kept in stables during autumn and winter.
**Azerbaijan**	10.110	4.806	6,1-**6,9**	> 50%	37%	**8.189** (0.81 hds/capita)	**10,2%**	82%	18%	Under development/ implementation	SR are grazed all summer on mountain pastures and pastures closer to the holdings in winter.	Markets are typically held on city outskirts with minimal infrastructure and oversight. Most are organised privately and lack legal supervision or veterinary controls.	Sheep and, to a lesser extent, goats play an important economic role in rural households. Animals are grazed on mountain pastures in summer and remain closer to the holdings during winter.
**Georgia**	3.714	4.698	7,8-**7,3**	50%	38%	**685** (0.18 hds/capita)	**0,9%**	95%	5%	Under development/ implementation	SR flocks are kept in lowlands in Autumn-Winter and mountains in spring-summer.	Live animal markets are officially registered and host multi-species trade on designated days. However, animal movements at markets are not officially recorded.	Agriculture is a key livelihood in rural areas, where small ruminants are typically grazed in lowlands during autumn and winter, and in mountainous areas during spring and summer.
**Belarus**	9.399	6.839	6,2-**6,8**	-	11%	**148** (0.015 hds/capita)	**0,2%**	91%	9%	Yes	No	SR are not traded in markets, although legal provisions exist for trade regulation, including biosecurity measures such as quarantine and diagnostics.	Small ruminant production holds limited socio-economic importance in Belarus. Legal regulations for animal trade exist but are rarely applied to SR due to minimal market presence.
**Bulgaria**	6.927	9.828	4,0-**3,4**	26%	11%	**2.015** (0.29 hds/capita)	**2,5%**	76%	24%	Yes	SR stay in pastures March-November and are kept indoors or in nearby fields in Winter. Movements are recorded in a national recording system.	Eight authorised livestock markets exist, regulated under national legislation. Despite veterinary oversight and movement certification, SR trade in markets remains limited.	SR production is concentrated in southern regions, particularly those bordering Türkiye. Sheep farming is traditional and plays a critical role in rural community livelihoods and regional development.

**Moldova**	**2.618**	**4.494**	**11,5-9,5**	**12%**	**27%**	**845 (0.32 hds/capita)**	**1,1%**	**87%**	**13%**	**Yes**	**No**	**Animal markets are regulated by national legislation, including rules for trade, transport, exhibitions, and similar events involving animals.**	**Small ruminant farming focuses on milk production. Goat milk and cheese are primarily for household consumption, while sheep milk cheese is both consumed and marketed.**
**Romania**	19.286	12.890	4,1-**3,8**	26%	21%	**12.541** (0.65 hds/capita)	**15,6%**	96%	4%	Yes	Yes	Since EU accession in 2007, Romanian livestock markets follow EU legislation for traceability and animal movements. Markets play an active role in formal SR trade.	Romania is among the top five European exporters of live sheep and goats, primarily to Middle Eastern and Southern European countries. Over 20% of livestock was exported in 2019.
**Türkiye**	84.339	9.127	6,8-**6,6**	25%	18%	**54.113** (0.64 hds/capita)	**67,3%**	93%	7%	Yes	Yes	Livestock markets are regulated regionally. All transactions are recorded under the national monitoring system (TURKVET), ensuring traceability and sanitary compliance.	SR production is essential for income generation, food security, employment, and limiting rural depopulation. Sheep and goats fulfil multiple roles in rural economies across the country.
**Ukraine**	44.135	3.663	12,0-**9,2**	-	14%	**1.144** (0.026 hds/capita)	**1,4%**	87%	13%	Yes	No	Ruminants are not commonly traded in livestock markets, which mainly serve other species such as poultry, rabbits, and swine.	Small ruminant production is mostly subsistence-based and led by rural households. These households account for the vast majority of national production of SR meat and milk.

GDP: Gross domestic product; hds: number of heads; M: thousand (Arede et al., 2023).

S2: Status and management practices in countries of the study region

Based on Legnardi et al., 2022. Azerbaijan was officially recognised by WOAH as PPR-free in 2024, whereas all the other countries in the study region are not recognised by WOAH as PPR-free (as of January 2025).

Country	LAST OUTBREAK	SURVEILLANCE	VACCINATION
Armenia	Never been reported	Active (risk-based)	Not applied
Azerbaijan	Never been reported	Active	Not applied
Belarus	Never been reported	Not included in the animal surveillance programme	Not applied
Bulgaria	2024 (First reported in 2018)	Active (risk-based in regions near Thrace); passive for the whole country	Not applied
Georgia	2024 (First reported in 2016)	Active	Yes
Moldova	Never been reported	Passive	Not applied
Romania	2024 (First PPR report)	Active and passive (clinical exams before and after pasture season)	Not applied
Türkiye	Anatolia: Endemic	Anatolia: passive	Anatolia: vaccination
	Thrace: Absent (PPR-protected area)	Thrace: active since March 2021	Thrace: not applied since March 2021
Ukraine	Never been reported	Passive	Not applied

Based on (Arede et al., 2023; Legnardi et al., 2022)

S3: Response to PPR outbreaks in Georgia (2016) and in Bulgaria (2018)

Besides for endemic Türkiye, PPR outbreaks were reported for the first time in the study region, in central Georgia in 2016 (Dundon et al., 2018) and southeast Bulgaria in 2018 (WAHIS, 2018). A swift containment of these outbreaks was achieved through effective control measures including epidemiological investigations, stamping out, delimitation of protection zones, and in Georgia, vaccination of all susceptible animals. Additionally, risk-based surveillance, awareness campaigns, and yearly vaccination of young SRs in Georgia continued through 2022 (Legnardi et al., 2022)**,** highlighting robust PPR management in these areas. However, in both cases, the exact sources of PPR introduction were not confirmed (de Clercq et al., 2018; Legnardi et al., 2022). In Bulgaria, the epidemiological investigation following the outbreak concluded that the high demand for mutton and the resulting price differences during religious festivals between Bulgaria and Thrace, contributed to increased informal movements of people and livestock across the border (de Clercq et al., 2018). In Georgia, molecular analyses indicated that the detected strain was more closely related to sequences from Northern and Eastern Africa than to those from neighbouring countries, suggesting an unknown epidemiological link (Donduashvili et al., 2018; Legnardi et al., 2022).

S4: Pairwise comparison matrix exercise

The pairwise comparison matrix of the analytical hierarchy process (AHP) for risk factors (RFs) associated with the spread of peste des petits ruminants (PPR) is shown below. This table illustrates the elicitation exercise (EE) created in Excel and sent to experts. The EE aimed to compare the relative importance of each pair of RFs, from the row RF to the column RF, as illustrated by the arrows. To do this, selected experts completed each cell indicating "Select a key value" with one expression from the key table shown below. The number equivalent to each expression was then used to calculate the RF weights, as further described in the next section (S4).

KEY

**Less important**				Equivalent				**More important**
Extremely	Very strongly	Strongly	Moderately		Moderately	Strongly	Very strongly	Extremely
$^1/_9$	$^1/_7$	$^1/_5$	$^1/_3$	1	3	5	7	9

Elicitation Exercise

Column RF / Row RF	**Sheep and goat density**	**Proximity to areas previously affected by PPR**	**Smallholder farming**	**Proximity to markets**	**Seasonal pastures**
**Sheep and goat density**		Select a key value	Select a key value	Select a key value	Select a key value
**Proximity to areas previously affected by PPR**			Select a key value	Select a key value	Select a key value
**Smallholder farming**				Select a key value	Select a key value
**Proximity to markets**					Select a key value
**Seasonal pastures**					

S5: Risk factor (RF) weight calculation steps

RF weights for each completed matrix were calculated based on analytical hierarchy process (AHP) calculation steps, adapted by Coyle, G (Coyle, 2004).

Having a pairwise comparison matrix with ***n*** number of criteria (4) and hypothetical attributed values. We calculated 1) the nth root for the product of each row; and 2) the normalized eigenvector: the quotient of the value calculated in 1) by their total.

For a specific matrix, the normalized eigenvector is the weight attributed to each criterion.

	**A**	**B**	**C**	**D**	**n^{th} root of the product of row values**	**Eigenvector**
**A**	1	1/3	1/9	1/5	0.293	0.058
**B**	3	1	1	1	1.316	0.262
**C**	9	1	1	3	2.279	0.454
**D**	5	1	1/3	1	1.136	0.226
**Total**					5.024	1

Then, we calculated the Consistency Index (CI) and Consistency Ratio (CR) to assess the consistency of each matrix.

- To achieve this, the lambda max (λmax) is calculated for each row, in two steps. First, we calculate the sum of products of the eigenvector and attributed value for each column and row, respectively (e.g.: Lambda max of 1st row, 1*0.058+1/3*0.262+1/9*0.454+1/5*0.226 = 0.240), which should be larger than ***n***. Second, we calculate the lambda max value by dividing the vector calculated previously by the eigenvector.

- The CI is calculated by:

$$CI=\frac{\lambda max\text{-}n}{n\text{-}1}$$

- The CR is calculated by the CI, using a random index based on the ***n*** and the values of CI of matrices filled in at random, from the following table.

	Matrix size (number of criteria)							
n	1	2	3	4	5	6	7	8
**RI**	0.00	0.58	0.90	1.12	1.24	1.32	1.41	1.45

$$CR=\frac{CI}{RI}$$

S6: Responses to the elicitation exercise (EE)

This graph presents the number of responses obtained from the EE, categorised as consistent (CR<0.14) and not consistent (CR≥0.14). National experts are subcategorised by their country of origin, and international experts are presented as a separate group.

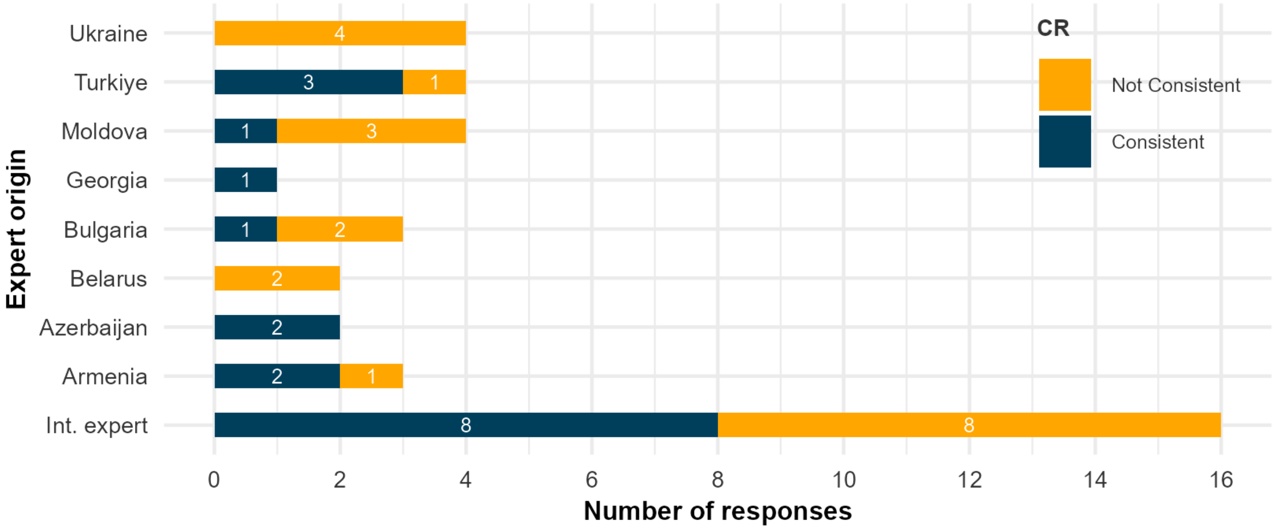

S7: Suitability maps for peste des petits ruminants (PPR) spread in the Black Sea basin (BSB) by expert group

The maps below illustrate the suitability maps for the spread of PPR in the BSB generated with the mean RF weights attributed by each expert group (national and international experts). The suitability index (SI) is displayed using a diverging spectral colour scale, categorised into ranges, where blue and green indicate low suitability, yellow and light orange indicate medium suitability, and darker orange and red indicate high suitability for PPR spread.

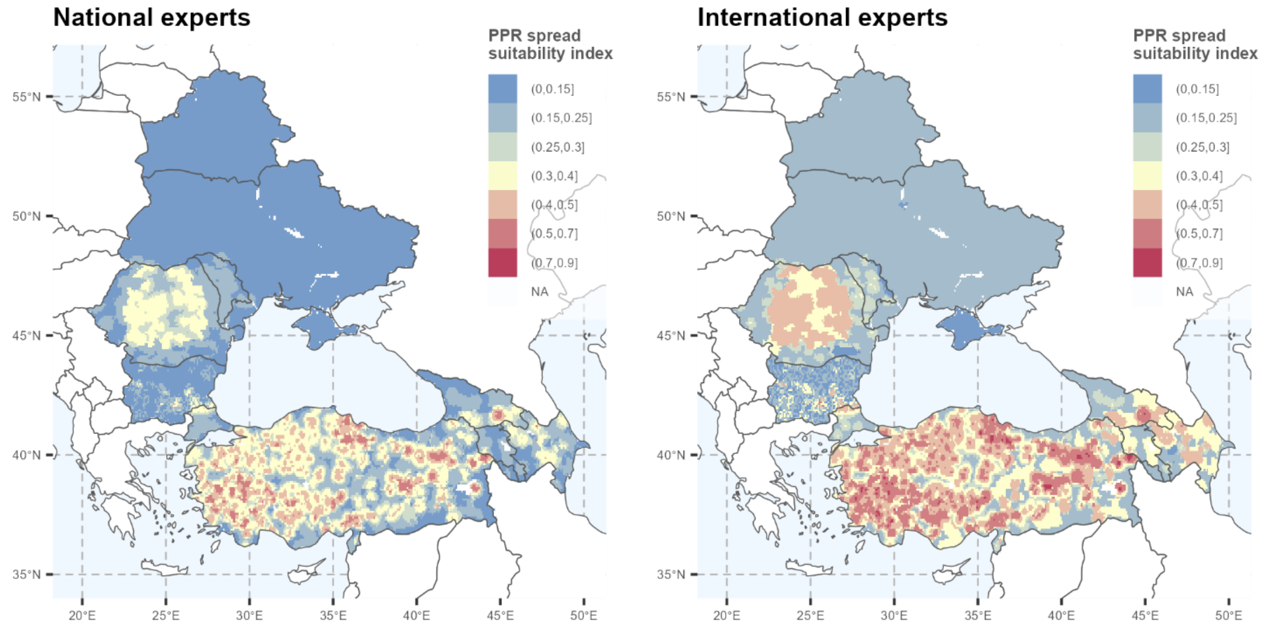

S8: The receiver operating characteristic (ROC) curve for the validation of suitability maps.

The graphs below present the ROC curves for the validation of suitability maps for peste des petits ruminants (PPR) spread based on the mean risk factor (RF) weights from all experts (A), international experts (B) and national experts (C). The validation was conducted using PPR outbreak locations from Türkiye notified between 2020 and 2021, along with generated pseudoabsences.

Total of experts (A)

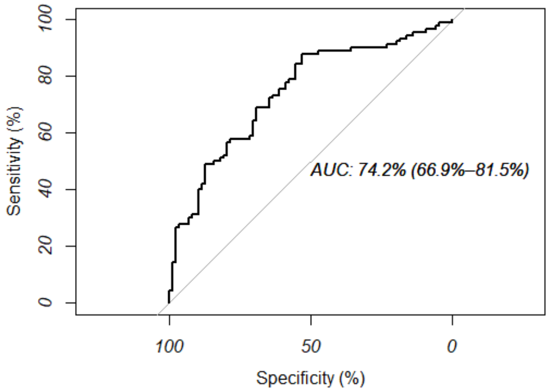

International experts (B)

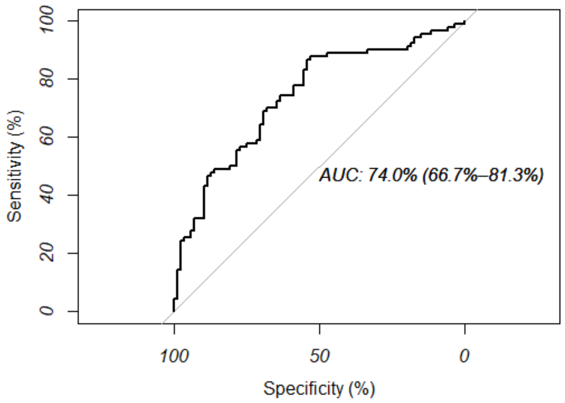

National experts (C)

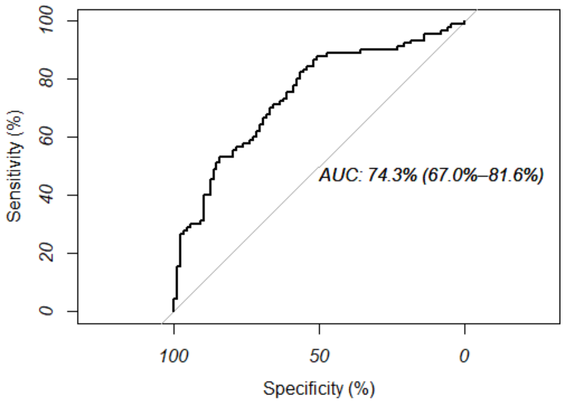

S9: Global sensitivity analysis of the PPR spread suitability map

The graph below shows the mean of absolute change rate (MACRs) for the suitability map of peste des petits ruminants (PPR) spread generated with mean risk factor (RF) weights from all experts. It depicts the MACR of the suitability for each RF (proportion of smallholder farms, small ruminant abundance, proximity to previous PPR outbreaks, seasonal movements, and proximity to livestock markets) change rate in a range of -25% to +25%. Each coloured line reflects how the suitability for PPR spread changes in response to weight changes in the respective RF.

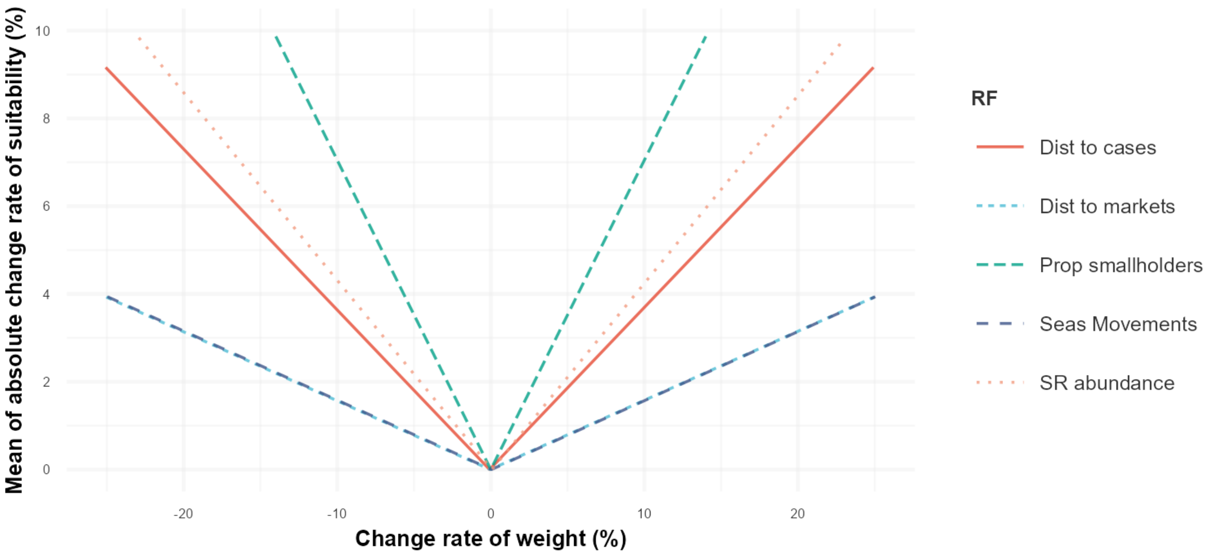

References

Arede, M., Beltran Alcrudo, D., Aliyev, J., Chaligava, T., Keskin, I., Markosyan, T., Morozov, D., Oste, S., Pavlenko, A., Ponea, M., Starciuc, N., Zdravkova, A., Raizman, E., Casal, J., Allepuz, A., 2023. Examination of critical factors influencing ruminant disease dynamics in the Black Sea Basin. Front. Vet. Med. 10. https://doi.org/10.3389/fvets.2023.1174560

Coyle, G., 2004. THE ANALYTIC HIERARCHY PROCESS (AHP).

de Clercq, K., Cetre-Sossah, C., Métras, R., 2018. Mission of the Community Veterinary Emergency Team to Bulgaria - PPR outbreak in 2018. European Commission (EC).

Donduashvili, M., Goginashvili, K., Toklikishvili, N., Tigilauri, T., Gelashvili, L., Avaliani, L., Khartskhia, N., Loitsch, A., Bataille, A., Libeau, G., Diallo, A., Dundon, W.G., 2018. Identification of peste des petits ruminants virus, Georgia, 2016. Emerg. Infect. Dis. 24, 1576–1578. https://doi.org/10.3201/eid2408.170334

Dundon, W.G., Donduashvili, M., Tigilauri, T., Gelashvili, L., Avaliani, L., Khartskhia, N., Loitsch, A., Bataille, A., Libeau, G., Diallo, A., Cattoli, G., 2018. Identification of Peste-des-petits ruminants, Georgia. Book Abstr. ESVV 2018.

Legnardi, M., Raizman, E., Beltran-Alcrudo, D., Cinardi, G., Robinson, T., Falzon, L.C., Djomgang, H.K., Okori, E., Parida, S., Njeumi, F., Benfield, C.T.O., 2022. Peste des Petits Ruminants in Central and Eastern Asia/West Eurasia: Epidemiological Situation and Status of Control and Eradication Activities after the First Phase of the PPR Global Eradication Programme (2017-2021). Anim. Open Access J. MDPI 12, 2030. https://doi.org/10.3390/ANI12162030

WAHIS, 2018. Animal Disease Events (PPR, Bulgaria). (World Animal Health Information System.
